# Dynamic Method of Neutral Axis Position Determination and Damage Identification with Distributed Long-Gauge FBG Sensors

**DOI:** 10.3390/s17020411

**Published:** 2017-02-20

**Authors:** Yongsheng Tang, Zhongdao Ren

**Affiliations:** 1 School of Urban Rail Transportation, Soochow University, Suzhou 215137, China; 2 Department of Civil Engineering, Yanshan University, Qinhuangdao 066004, China; zhongdaoren96@163.com

**Keywords:** neutral axis position, dynamic macro-strain, modal macro-strain, long-gauge FBG sensors, structural performance assessment

## Abstract

The neutral axis position (NAP) is a key parameter of a flexural member for structure design and safety evaluation. The accuracy of NAP measurement based on traditional methods does not satisfy the demands of structural performance assessment especially under live traffic loads. In this paper, a new method to determine NAP is developed by using modal macro-strain (MMS). In the proposed method, macro-strain is first measured with long-gauge Fiber Bragg Grating (FBG) sensors; then the MMS is generated from the measured macro-strain with Fourier transform; and finally the neutral axis position coefficient (NAPC) is determined from the MMS and the neutral axis depth is calculated with NAPC. To verify the effectiveness of the proposed method, some experiments on FE models, steel beam and reinforced concrete (RC) beam were conducted. From the results, the plane section was first verified with MMS of the first bending mode. Then the results confirmed the high accuracy and stability for assessing NAP. The results also proved that the NAPC was a good indicator of local damage. In summary, with the proposed method, accurate assessment of flexural structures can be facilitated.

## 1. Introduction

Flexural structures such as beams are the most common structures in civil engineering. However, they are often damaged due to severe environment, over loading, original structural flaws, earthquake, typhoon or other factors. Therefore, it is important to ensure the safety of these structures. For addressing this issue, the concept of structural health monitoring (SHM) has been proposed and widely studied for structural maintenance and management in the last 30 years [1,2,3,4]. In the SHM systems, some key structural parameters should be selected to be monitored and assessed.

The neutral axis is the axis about which bending occurs in a beam or a composite section. As a key parameter, the neutral axis position (NAP) is so important that it is needed in most theories of structural design. Moreover, the neutral axis position serves as a potential indicator of the structure’s safety condition. For instance, Griffin et al. [5] measured the neutral axis locations of two strengthened bridges, from which the retrofitting effects were verified as the neutral axis moved as expected after reinforcement.

Many other researchers have already investigated how to determine the neutral axis position from static or live traffic loading tests [6,7,8,9,10,11,12,13]. The static loading test generally costs much labor and time, and it is not suitable for long-term structural monitoring. Therefore, attention has been focused on live traffic loading tests for neutral axis position determination. Gutiérrez et al. [6] identified the neutral axis location of a bridge beam with reinforced concrete decks and carbon Fiber reinforced polymer (CFRP) box girders directly using the dynamic strain data. The results showed that the identified neutral axis position fluctuated very much especially when the excitation is not strong enough. Elhelbawey et al. [7] evaluated the neutral axis locations by using the static and dynamic strains measured at the girder’s bottom, middle and top with four methods. There were obvious variations in results from different methods. Chakraborty and Dewolf [8] estimated the neutral axis locations through the traffic loading test, the results of which also showed large variations when using different time windows of the test data. Gangone et al. [9] obtained the neutral axis location with strains measured by wireless strain sensors under live truck loading, which passed the bridge at about 16.1~24.1 km/h. The neutral axis location was found to have a strong relationship with the loading position. Similar work has also been performed with a conclusion that the dynamic effect was a likely reason to induce the result variation [10,11].

It is seen from the literature review that the biggest problem in determining the neural axis position from the dynamic strain is that the results have obvious variations. Different explanations have been brought forward: (1) the uncertainty for strain measurements is obvious especially when the absolute value of the measured strain is small [9]; (2) force types influence the strain distribution along the cross section, such as dynamic effects, torsion effects and axial forces [8]; (3) the loading magnitude and loading location change the composite action between the girders and the deck, which affects the neutral axis position [7]; and (4) nonlinear structure performance influences the neutral axis location as well when large strain occurs [12,13]. Researchers have also proposed strategies to improve the accuracy of the neural axis position estimation: (1) the maximum strain was used to evaluate the neutral axis location as it was considered to be more reliable [8]; (2) the average value was selected [9]; (3) the root-mean square (RMS) value was proposed with a relatively good stability [7].

Some improvements have indeed been achieved with these methods. However, reliability and accuracy of the neural axis position assessment using dynamic strain still needs further exploration. Therefore, a new method is developed to determine the neutral axis position in this paper. In the proposed method, the dynamic strain is first acquired with the long-gauge macro-strain sensors, considered as a useful way to detect local damage. Then the modal macro-strain (MMS) is generated from strain time histories and is used to calculate the neutral axis location. The proposed method has the merit of high stability as it uses the MMS which is robust to loading situations and noises. Finally, some experiments are conducted to verify the effectiveness of the proposed method. 

## 2. Dynamic Method of Neutral Axis Position Determination

### 2.1. Strain Measurement with Long-Gauge Fiber Optic Sensors

**Figure 1:**
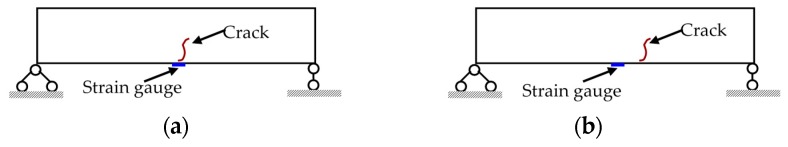
Strain measurement with strain gauge: (**a**) strain gauge locates at the crack; and (**b**) strain gauge locates at some distance away from the crack.

There are many methods to measure strain, of which the strain gauge is the most common type of sensors. Usually the gauge length is only several centimeters, so the measurement is often named as point measurement. It means that the strain reflects only the deformation at some point on the monitored structure. It may not easy to locate some local damage directly with the strain gauge, such as concrete crack as shown in Figure 1. In actual measurement the strain gauge hardly locates at the same position due to the random distribution of the damage, so it usually needs some additional complicated techniques to locate the damage.

**Figure 2:**
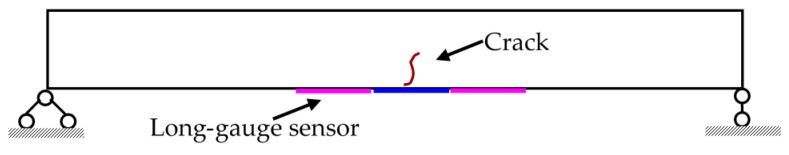
Area sensing with long-gauge sensors.

Compared with the point sensing, area sensing is proposed along with application of long-gauge sensors [14]. The parameter measured by the long-gauge sensor is the average strain of the gauge length, named as macro-strain. The gauge length can be up to 1 m ~ 2 m, much larger than that of the common strain gauge. Then the damage information can be obtained by the measured macro-strain if the damage locates within the sensor. The probability to capture the damage is greatly increased when several long-gauge sensors are applied at the same time (Figure 2) because the monitored area can be enlarged to cover the entire area.

**Figure 3:**
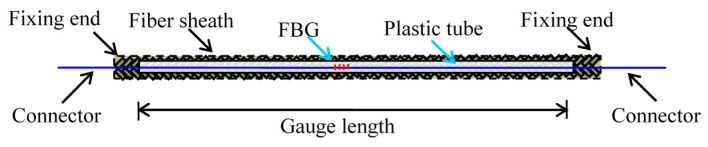
Packaged long-gauge Fiber Bragg Grating (FBG) sensor.

Due to its excellent accuracy, high data acquisition speed and the multiplexing performance, the Fiber Bragg Grating (FBG) sensor is used to develop the long-gauge macro-strain sensor (Figure 3). Every point in the gauge length presents the same mechanical behavior and hence the strain obtained from FBG can represent the average strain over the gauge length, termed as macro-strain. To actualize the long-gauge concept for real sensors, the FBG sensor is fixed at the two ends of a plastic tube surrounded by a fiber sheath impregnated with epoxy resin. The diameter of the packaged sensor is about 1 mm. Meanwhile, the gauge length can be set from 0.1 m to 2 m.

### 2.2. Macro-Strain Modal Analysis

Like the point strain, the macro-strain measurement can also be used for strain modal analysis and extraction of the macro-strain mode [15].

**Figure 4:**
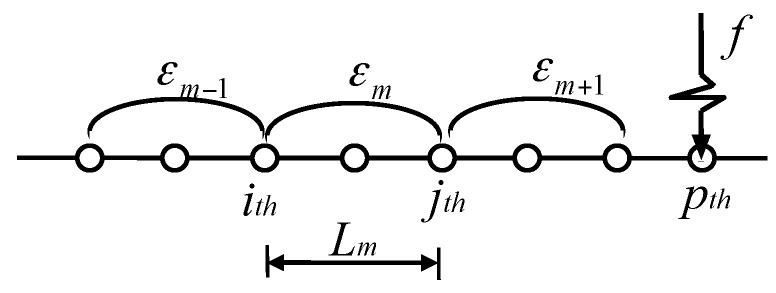
Macro-strain measurements for beam structure.

In Figure 4, a typical beam model is taken as an example. The strain *ε_m_* is measured by the strain sensor covering the zone from the *i_th_* node to *j_th_* node, namely, the gauge length *L_m_*, when force *f* is applied at the *p_th_* node. The *ε_m_* can be expressed as

(1) $$\varepsilon_{m}=\frac{h_{m}}{L_{m}}\left(v_{j}-v_{i}\right)=\eta_{m}\left(v_{j}-v_{i}\right)$$

Then, the equation in the frequency domain is

(2) $$\varepsilon_{m}(\omega )=\eta_{m}\left[v_{j}(\omega )-v_{i}(\omega )\right]$$

In Equations (1) and (2), *v_i_* and *v_i_* (*w*) are the rotational displacement for the *i_th_* node in the time and frequency domain, respectively. *h_m_* is the distance from the strain sensor to the neutral axis.

Frequency response function (FRF) is considered to be related with structure properties only. For the macro-strain FRF $H_{mp}^{\varepsilon }(\omega )$, it can be achieved by

(3) $$H_{mp}^{\varepsilon }(\omega )=\frac{\varepsilon_{m}(\omega )}{f_{p}(\omega )}=\frac{\eta_{m}\left[v_{j}(\omega )-v_{i}(\omega )\right]}{f_{p}(\omega )}=\eta_{m}(H_{jp}^{d}(\omega )-H_{ip}^{d}(\omega ))$$

The $H_{ip}^{d}(\omega )$ is the traditional rotational displacement FRF, which can be expressed as

(4) $$H_{ij}^{d}(\omega )=\sum_{r=1}^{N}\frac{\varphi_{ir}\varphi_{jr}}{M_{r}(\omega_{r}^{2}-\omega^{2}+2i\xi_{r}\omega_{r}\omega )}$$

where $\varphi_{ir}$ is the *r_th_* rotational displacement mode at the *i_th_* node. *M_r_* and *ω_r_* are the mass and resonant frequency for the *r_th_* order mode, respectively. $\xi_{r}=C_{r}/\left(2M_{r}\omega_{r}\right)$, while *C_r_* is damping efficient for the *r_th_* mode. Finally, the macro-strain FRF can be expressed as

(5) $$H_{mp}^{\varepsilon }(\omega )=\sum_{r=1}^{N}\frac{\eta_{m}(\varphi_{jr}-\varphi_{ir})\varphi_{pr}}{M_{r}(\omega_{r}^{2}-\omega^{2}+2i\xi_{r}\omega_{r}\omega )}=\sum_{r=1}^{N}\frac{\delta_{mr}\varphi_{pr}}{M_{r}(\omega_{r}^{2}-\omega^{2}+2i\xi_{r}\omega_{r}\omega )}$$

Compared with Equation (4), the similarity is found in Equation (5) except parameter *δ_mr_*.

The absolute value at the peak for the *r_th_* order mode can be written as

(6) |Hmpε¯r(ω=ωr)|=|φpr2Mrξrωr2δmr|

Here, for a given mode and a given excited point, $\varphi_{pr}/(2M_{r}\xi_{r}\omega_{r}^{2})$ is a constant. Therefore, *δ_mr_* is the parameter determining the macro-strain mode shape. 

As is well known, using the Fourier Transform (FT), the relationship can be obtained at the *r_th_* order mode as

(7) $$\frac{\varphi_{ir}}{\varphi_{jr}}=\frac{v_{i}(\omega_{r})}{v_{j}(\omega_{r})}$$

It is written to be as Equation (8) for the macro-strain mode.

(8) $$\frac{\delta_{mr}}{\delta_{nr}}=\frac{\varepsilon_{m}(\omega_{r})}{\varepsilon_{n}(\omega_{r})}$$

where *ε_m_*(*w_r_*) is the modal macro-strain (MMS) for the *m_th_* monitored element at the *r_th_* mode, namely the absolute value of the magnitude in the frequency spectrum when the frequency is *w_r_*.

Therefore, the macro-strain modal analysis can be implemented as the traditional displacement modal analysis using the macro-strain measurements.

### 2.3. Neutral Axis Position Determination with MMS

**Figure 5:**
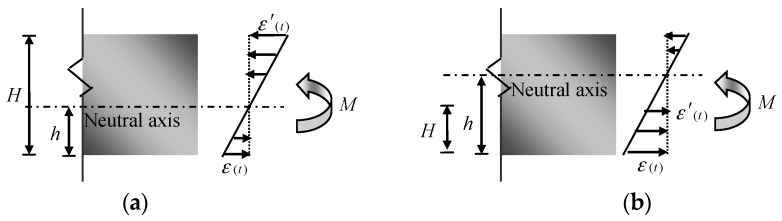
Strain distribution along the cross section with sensors installed at: (**a**) different sides of the neutral axis; and (**b**) the same side of the neutral axis.

As shown in Figure 5, the plane section assumption is used in each beam-like structure. It means that there are linear relationships among the strains along the section depth which are determined by the structure property itself. Based on this assumption, the neutral axis position coefficient (NAPC) *ψ* is applied to express the linear relationship

(9) $$\varepsilon (t)=\psi \varepsilon^{\prime }(t)$$

where ε(t) and $\varepsilon^{\prime }(t)$ are the strains at different depths in the same section. Besides the section, it is easily understood that the plane section can be also applied to analyze some small part of the flexural structure. In other words, the strain distributes linearly with an averaged neutral axis for all parts. Then the strains ε(t) and $\varepsilon^{\prime }(t)$ here are the macro-strains.

The neutral axis depth *h* (Figure 5) can be easily obtained by some basic geometrical knowledge. There are two cases for the strain measurement: (1) sensors installed at different sides of the neutral axis; and (2) sensors installed at the same side of the neutral axis. For these two cases, the equations to calculate the neutral axis depth are Equations (10) and (11), in which *H* means the vertical distance between the two sensors.

(10) $$h=\frac{\psi }{\psi +1}H$$

(11) $$h=\frac{\psi }{\psi -1}H$$

NAPC should be first solved to determine the neutral axis location. In Equations (12) and (13), with the Fourier transform (FT), the NAPC can be expressed by the modal macro-strain (MMS) $\varepsilon (w_{r})$.

(12) $$\varepsilon (\omega )=\int_{-\infty }^{+\infty }\varepsilon (t)e^{-i\omega t}dt=\int_{-\infty }^{+\infty }\psi \varepsilon^{\prime }(t)e^{-i\omega t}dt=\psi \int_{-\infty }^{+\infty }\varepsilon^{\prime }(t)e^{-i\omega t}dt=\psi \varepsilon^{\prime }(\omega )$$

(13) $$\psi =\frac{\varepsilon (w_{r})}{\varepsilon^{\prime }(w_{r})}$$

The NAPC *ψ* contains enough spatial information of the neutral axis. If damage occurs within the monitored zone, the neutral axis will move and the NAPC *ψ* will change as well. Therefore, it has the potential to be a new index to detect structural damage.

In this paper, only the first bending mode is applied to implement the method as it can be easily and accurately identified. Moreover, in the first bending mode, the structure is in a perfect bending state, improving the accuracy of the proposed method by ignoring the influence from some factors, such as torsion. Most of the measurement noise is also naturally filtered during the modal analysis process with the Fourier transform. Lastly the macro-strain mode is of no relationship with the loading magnitude and position and hence the MMS based method is not influenced by the loading. Therefore, compared with the traditional methods based on the strain in time domain, the proposed method offers a better assessing performance.

## 3. Verification with FE Models

### 3.1. FE Model Description

**Figure 6:**
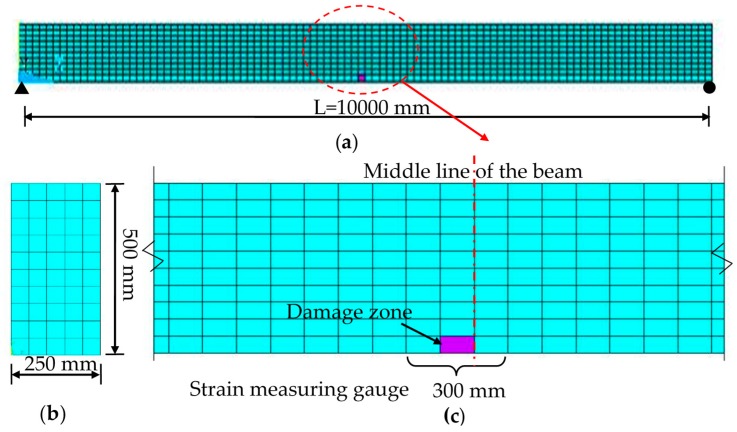
Finite Element (FE) beam model: (**a**) beam; (**b**) cross section; and (**c**) damaged zone and monitored zone.

Solid45 was selected to establish simply-supported Finite Element (FE) beam model with ANSYS. The detailed model information is shown in Figure 6. From the direction along the beam length, the element length was 100 mm, while it was 50 mm for the other two directions. The five elements along the width at the middle span, shown in Figure 6c, were selected as damaged elements by changing the elastic modulus. The damage cases are listed in Table 1, where ‘Stiffness loss’ only means the relative stiffness loss of the damaged elements. In the experiments the strain measuring gauge length was 300 mm, 3 times as long as the element length, completely covering the damaged zone. The dynamic loads were implemented at the point on the beam up-surface.

### 3.2. Neutral Axis Position

Through modal analysis, the MMS can be obtained with Equation (6). In this paper, only the first bending mode is used to do structural analysis. Then with the MMS of the upside element and the downside element, the neutral axis position can be determined with Equation (13). The results of neutral axis position coefficient (NAPC) were listed in Table 2. For comparison, the results of NAPC with static analysis were also listed in the table. From these results, the proposed dynamic method is verified with a good accuracy as the largest relative deviation between the modal analysis and static analysis is less than 1%. 

### 3.3. Damage Identification

As a new index to assess damage, damage sensitivity of the NAPC should be investigated and compared with some acknowledged indices, such as resonant frequency, strain mode and curvature mode. In Table 3, the relative change of the above four indices and neutral axis depth have been obtained from the simulation results with different damage cases. The results show the largest relative changes for strain mode. In other words, the strain mode method presents the highest damage sensitivity. Then it is the proposed NAPC, close to the value of the strain mode. The NAPC results are calculated with the MMS from the down-surface and up-surface. The damage sensitivity of the NAPC is much better than the curvature mode, which is accepted as an index sensitive to local damage. The neutral axis depth is not as sensitive as the NAPC, but it is still much more sensitive than the resonant frequency as the resonant frequency hardly changes under the simulated local damage. Moreover, compared with the strain mode and curvature mode methods, there is no need to select an intact referenced element for the proposed method. If some damage happens within the referenced element, the methods may fail to implement damage identification. Therefore, the proposed NAPC is a promising indicator to identify local damage, such as a concrete crack.

The factors influencing the damage sensitivity are also investigated with the FE modeling results. In the proposed method the sensor gauge length and sensor installation position are the two main factors. It can be easily understood that the smaller the sensor gauge is, the higher is the damage sensitivity if the sensor can cover the damage zone. Therefore, the influence from the sensor installation not sensor gauge length is only studied.

**Figure 7:**
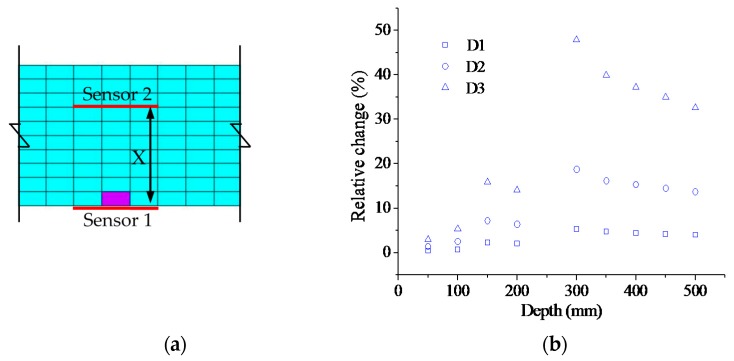
Damage sensitivity with different spatial sensor installations: (**a**) sensor installation; and (**b**) relative change of neutral axis position coefficient (NAPC).

As shown in Figure 7a, the distance “X” between the two sensors is taken as the variable to investigate the damage sensitivity of the NAPC. As the sensor can be easily installed on the beam bottom, Sensor 1 is considered fixed. The results are shown in Figure 7b. The strain is too small near the neutral axis, so the “X” of 250 mm was not investigated as the neutral axis location was in the range from 210 mm to 290 mm under the damage cases. From the results it is found that a much higher sensitivity can be obtained when the two sensors are installed on different sides of the neutral axis.

### 3.4. Performance under Noise

**Figure 8:**
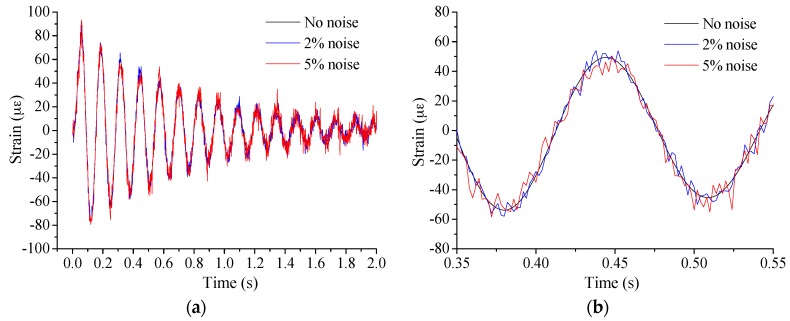
Typical strain results at Case I: (**a**) the whole time history; and (**b**) local part focused.

**Figure 9:**
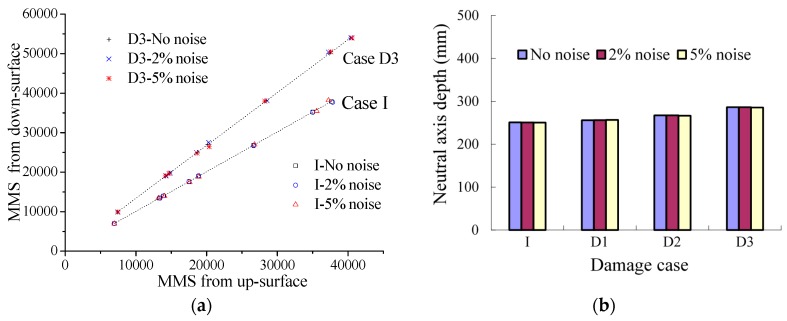
Performance of the proposed method under noise: (**a**) NAPC; and (**b**) neutral axis depth.

**Figure 10:**
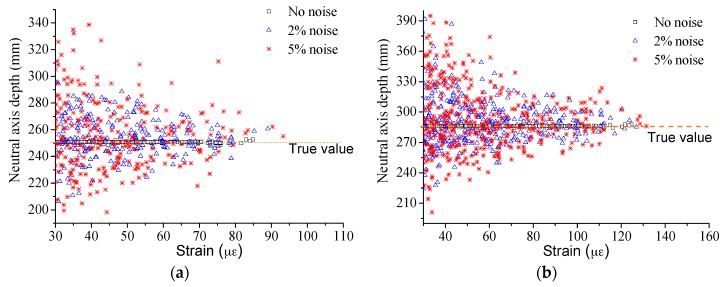
Performance of the traditional method under noise at Case: (**a**) I; and (**b**) D3.

Performance under noise is another issue to be investigated. As shown in Figure 8, the white noise was input into the strain results for investigation. The results of the proposed method are shown in Figure 9. The conclusion is that the added noise shows no influence on the proposed method even when the noise is up to 5%. However, the results will be influenced greatly by the noise when the strain is directly used to calculate the neutral axis position with the traditional method as Equation (9). Strain larger than 30 με was only applied in the calculation to reduce the assessment error caused by the strain measurement accuracy. From the results in Figure 10, the traditional method can hardly be implemented to assess the neutral axis position even with 2% noise, while it works well without noise. Therefore, the proposed method is a perfect choice to be applied in neutral axis position measuring under live traffic loads especially.

## 4. Verification with a Steel Beam

### 4.1. Experiment Description

**Figure 11:**
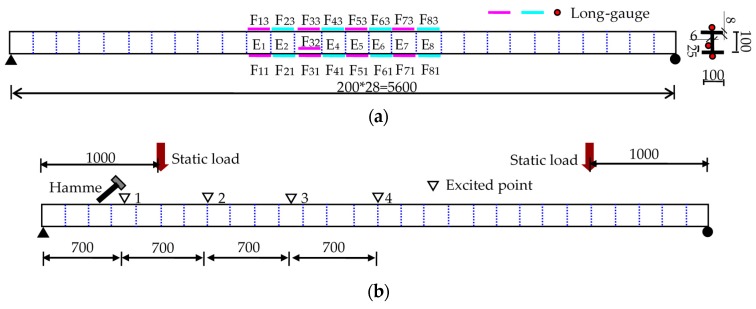
Experimental description for the steel beam tests (Unit: mm): (**a**) sensor installation at steel beam; and (**b**) loading position.

**Figure 12:**
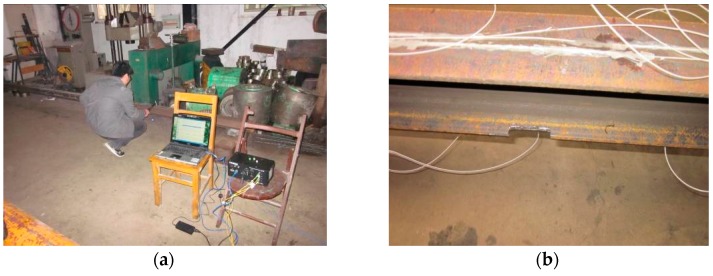
The steel beam experiment field: (**a**) dynamic test and data logger; and (**b**) damage simulation.

A steel beam (Figure 11) was prepared for some tests to verify the assessment accuracy of the proposed method. Considering the experience from the above RC beam tests, the damage was simulated by cutting some parts away from the downside flange as shown in Figure 12, guaranteeing the same neutral axis location during the dynamic and static tests.

In Figure 11a, the steel beam is 5.6 m long with an “I” type cross section, divided into 28 elements with an element length of 200 mm. The 8 elements, E_1_ to E_8_, were selected as monitored parts with long-gauge FBG sensors bonded on both up-surface (F_13_ to F_83_) and down-surface (F_11_ to F_81_). One more sensor, F_32_, was installed on the beam side surface to verify the plane section hypothesis. The reflected wavelength of FBG ranges from 1525 nm to 1580 nm with the sensitivity of about 1.2 pm/με.

In Figure 11b, static load was applied with some steel block, while the dynamic force was input by using the hammer to knock at the beam’s top surface. Besides the four points in the figure, the hammer was also used to knock at some random positions at one measure time to simulate the random dynamic cases. During the dynamic tests the strain was measured by SM-130 (Micron Optics Inc., Atlanta, GA, USA) with a sampling frequency of 1000 Hz as shown in Figure 12a. Table 4 presents the damage cases, D1 to D8, where the damage quantity is the relative loss of the element flexural-stiffness.

### 4.2. Experiment Results

**Figure 13:**
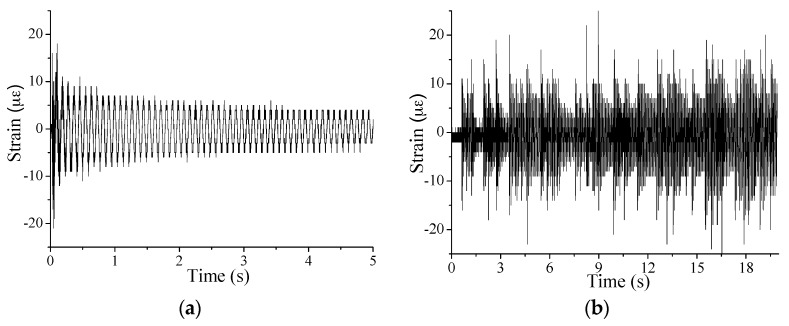
Typical macro-strain results for the steel beam test under: (**a**) single-point excitation; and (**b**) random excitation.

**Figure 14:**
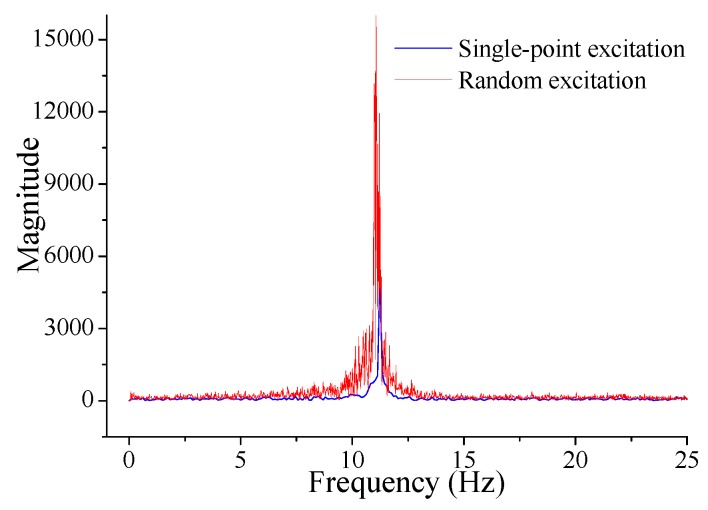
Typical results of macro-strain based frequency spectrum for the steel beam test.

Figure 13 presents the typical macro-strain results under the single-point and random excitation, while the macro-strain based frequency spectrum near the first bending mode is obtained with FFT to get MMS to implement the proposed method (Figure 14).

**Figure 15:**
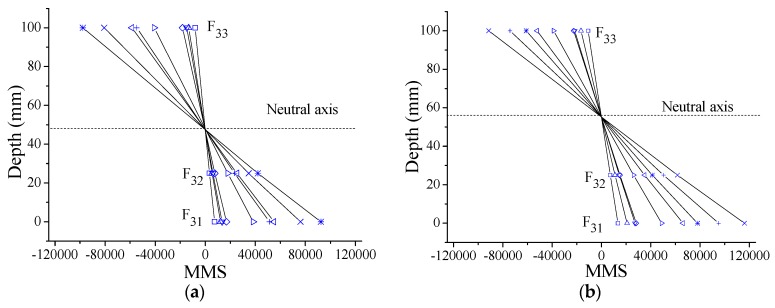
Modal macro-strain (MMS) distribution along the depth of the element E_3_ of the steel beam at Case: (**a**) I; and (**b**) D8.

**Figure 16:**
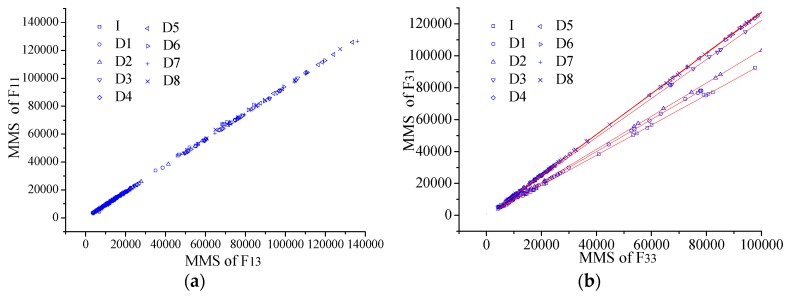
NAPC extracting of the steel beam from dynamic tests for: (**a**) E_1_; and (**b**) E_3_.

**Figure 17:**
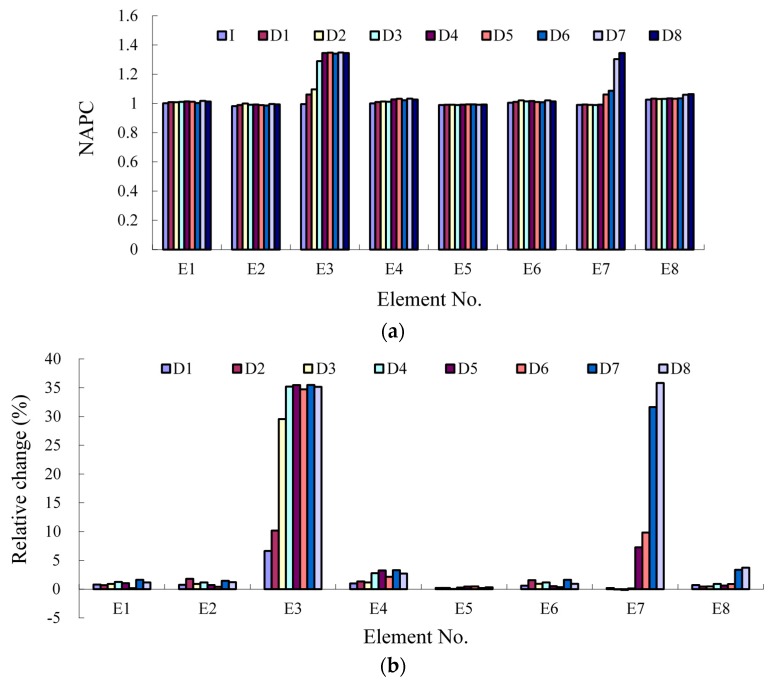
NAPC results of the steel beam: (**a**) absolute value; and (**b**) relative change.

With the obtained MMS, the plain section is verified at first (Figure 15). Then the NAPC is extracted from the results (Figure 16). In the figures all the linear correlation coefficients are larger than 0.99, indicating excellent stability of the NAPC obtained with the proposed dynamic method. The NAPC results are included in Figure 17 for all the monitored elements. From the relative change in Figure 17b especially the damaged elements can be detected easily even when the element stiffness loss is only 3%, proving the NAPC is an effective index to identify local damage.

With the obtained NAPC the neutral axis depth was calculated (Table 5). For the undamaged elements except E_4_ and E_8_, the relative measurement change is smaller than 1%, verifying the effectiveness of the proposed method. However, it is a little larger for E_4_ and E_8_, about 2%. The process of cutting steel parts to make damage caused some small damage within the two elements.

Then the results from the dynamic method are compared with the results from the static tests, as shown in Table 6. From the results, the largest absolute value of the relative difference is 2.6% for E_5_ at D5. It means that the relative error is less than 3% for the proposed dynamic method. Thus the high accuracy has been verified for assessing the neutral axis position with the proposed method. 

## 5. Verification with a Reinforced Concrete Beam

### 5.1. Experiment Description

**Figure 18:**
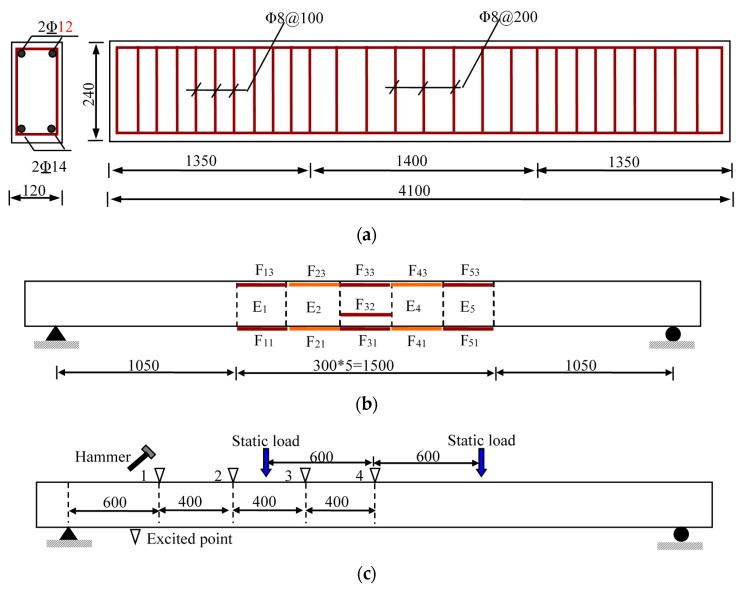
Experiment setup (Unit: mm): (**a**) reinforced concrete (RC) beam; (**b**) long-gauge FBG sensor distribution; and (**c**) loading positions.

One reinforced concrete (RC) beam as shown in Figure 18a is prepared to investigate the actual performance of the proposed method. The total beam length is 4100 mm, while the length between the two supports is 3600 mm. The rectangle section’s width is 120 mm, while the depth is 240 mm. The section is reinforced with two steel rebars with a diameter of 14 mm at downside and two of 12 mm at upside. Some other detailed information can be found in Figure 18a.

**Figure 19:**
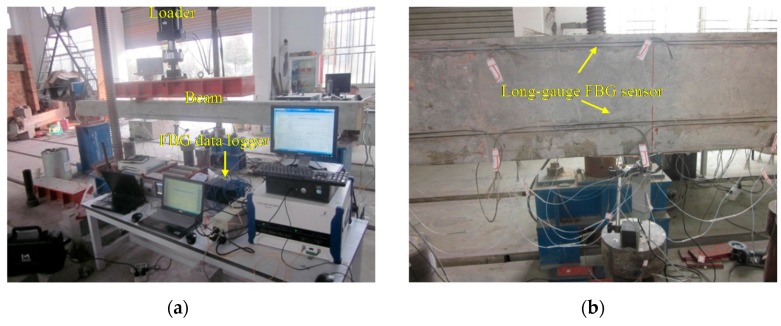
Experiment field: (**a**) beam specimen, loader and data logger; and (**b**) long-gauge FBG sensors.

In Figure 18b and Figure 19b, long-gauge FBG sensors (Figure 3) were installed to measure the macro-strain. F_11_~F_51_ were bonded at the beam bottom, while F_13_~F_53_ were at the beam side 20 mm away from the top. One more sensor F_32_ was installed at the beam side of the monitored element E_3_, 60 mm away from the bottom to prove the plane section. The gauge length is 300 mm, same for all the 11 sensors. The data logger is SM-130 made by Micron Optics Inc. (Atlanta, GA, USA) with a sampling frequency of 1000 Hz applied in the tests as shown in Figure 19a.

In Figure 18c, static load was applied to cause some structural damage, while the dynamic force was input by using the hammer to knock at the beam’s top surface. Besides the four points in the figure, the hammer was also used to knock at some random positions at one measure time to simulate the random dynamic cases.

**Figure 20:**
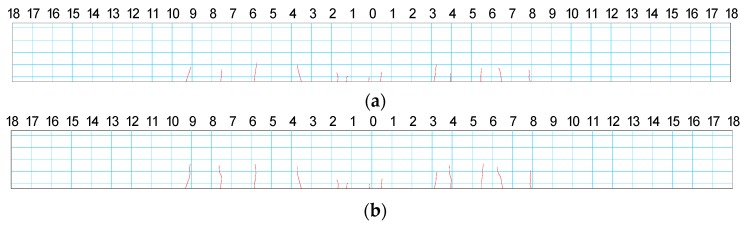

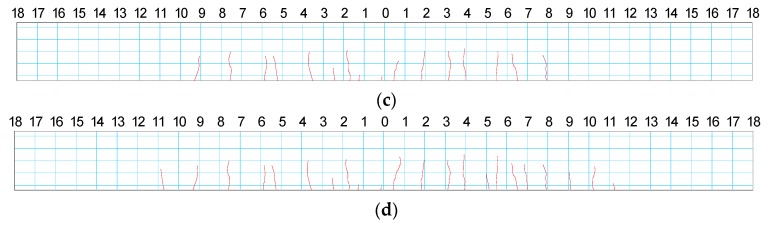
Concrete crack distribution at Case: (**a**) D1; (**b**) D2; (**c**) D3; and (**d**) D4.

The experiments were implemented in the following five cases: (1) Case I, dynamic test for the intact beam; (2) Case D1, static loading up to concrete cracking and unloading, then dynamic test; (3) Case D2, static loading to make concrete crack up to 1/4 beam depth and unloading, then dynamic test; (4) Case D3, static loading to make concrete crack up to 1/2 beam depth and unloading, then dynamic test; and (5) Case D4, static loading to make the monitored macro-strain up to 1000 με and unloading, then dynamic test. The actual concrete crack distribution is shown in Figure 20, in which the cracks were identified by visual inspection and measured by rules from one side of the beam. Due to the difficulty to induce the initial damage, the damage is much larger than the plan for Case D1, while the other three cases are implemented as per the plan.

### 5.2. Experiment Results

#### 5.2.1. Macro-Strain and Macro-Strain Based Frequency Spectrum

**Figure 21:**
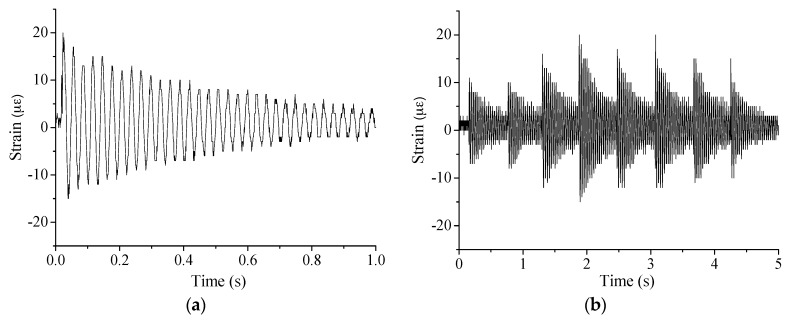
Typical macro-strain results under: (**a**) single-point excitation; and (**b**) random excitation.

**Figure 22:**
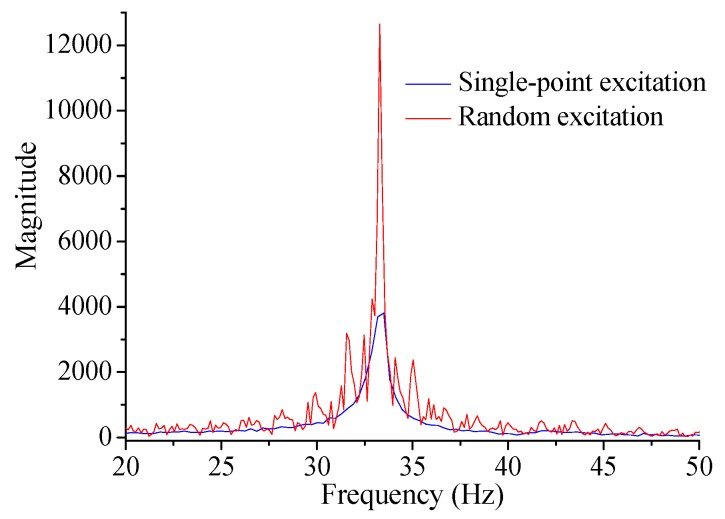
Typical results of macro-strain based frequency spectrum.

Figure 21 presents the typical dynamic macro-strain results under the single-point excitation and random excitation. To extract the modal information, the measured dynamic macro-strain was first transformed into frequency domain using the classic fast Fourier transform (FFT) method. In Figure 22, the typical results are obtained near the first bending mode frequency, 33.25 Hz, where the magnitude peak value is the MMS for the measurement. Then the proposed method can be applied to extract the information of the neutral axis position.

#### 5.2.2. Verification of Plane Section Hypothesis

**Figure 23:**
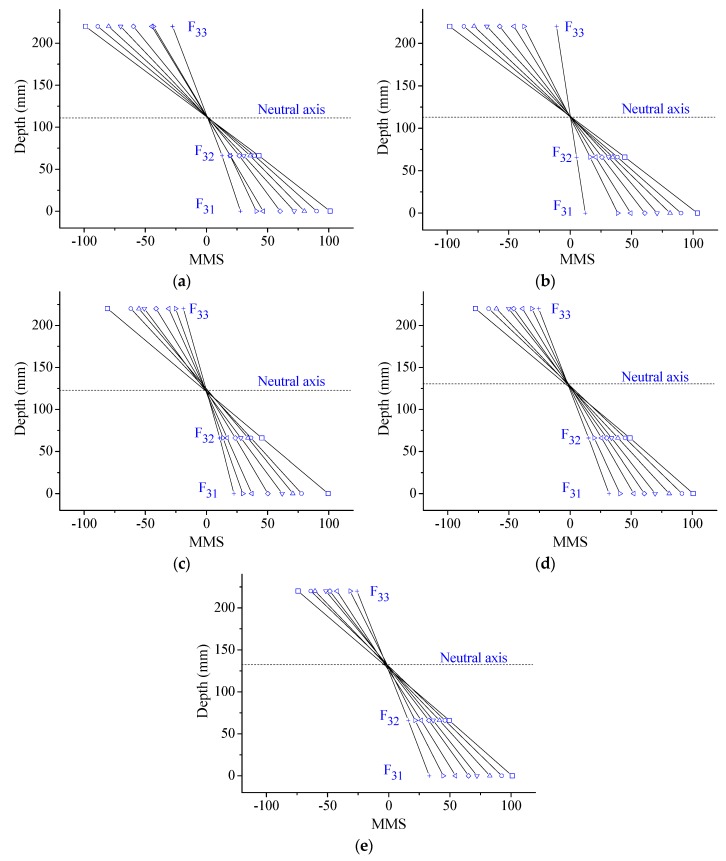
MMS distribution along the depth of the element E_3_ at Case: (**a**) I; (**b**) D1; (**c**) D2; (**d**) D3; and (**e**) D4.

It is easy to theoretically prove the plane section with the MMS as Equation (12). However, it is most important to implement the verification of the plane section hypothesis with the experimental MMS results. From the results of the element E_3_ in Figure 23, the MMS distributes linearly at all the cases with one rotation center where the MMS value is zero. Therefore, the plane section has been verified when the MMS is applied. Moreover, the neutral axis lies at the rotation center, as per the definition. From the results in Figure 23, it is obvious that the neutral axis is moving up step by step with loading.

#### 5.2.3. Neutral Axis Position Coefficient

**Figure 24:**
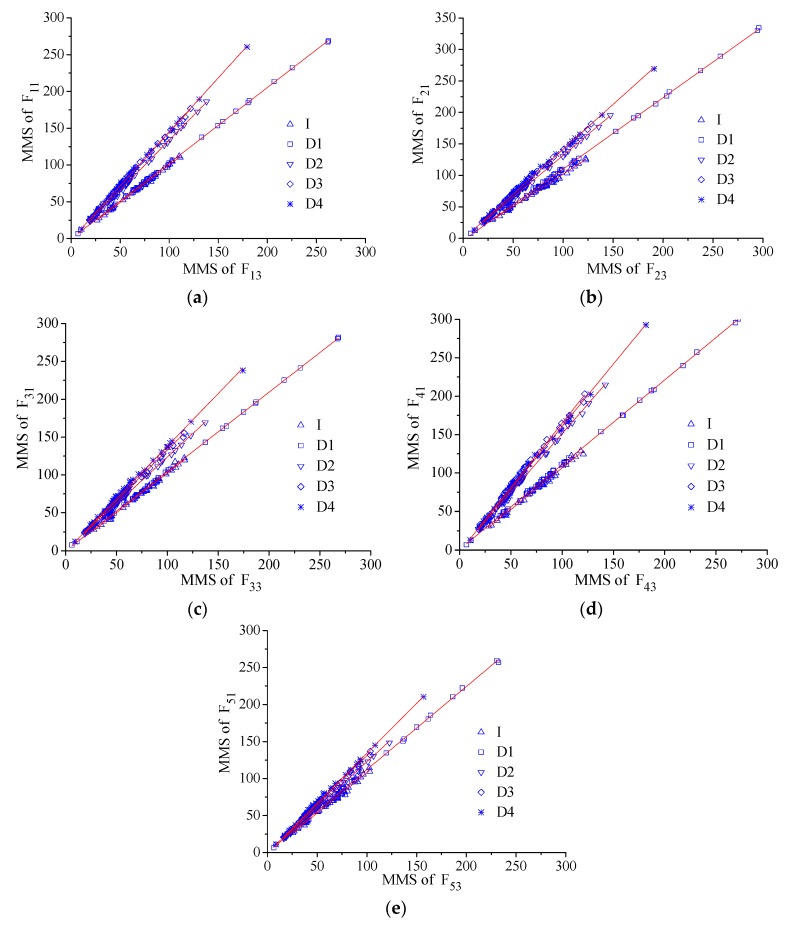
NAPC extracting from dynamic tests for: (**a**) E_1_; (**b**) E_2_; (**c**) E_3_; (**d**) E_4_; and (**e**) E_5_.

As shown in Figure 24 the line was fit between the MMSs from the two sensors at up-surface and down-surface (e.g., long-gauge FBG sensors F_31_ and F_33_ for E_3_ as shown in Figure 18b) to obtain the neutral axis position coefficient (NAPC) *ψ*. The fitting line slope is just the NAPC, increasing obviously with the damage developing in the figures. The perfect linear correlation has also been found from all the results as all the fitting correlation coefficients are larger than 0.99. In other words, it has been proved that the new index NAPC presents excellent stability, which is of great importance for long-term SHM. 

**Figure 25:**
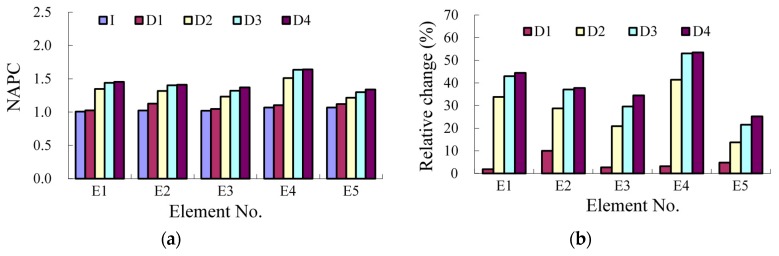
Results of NAPC: (**a**) absolute value; and (**b**) relative change.

In Figure 25a and Table 7, results of NAPC for all the five monitored elements are included, for all the cases. At Case I the NAPC of all the five elements are close to each other as there is no damage happening, while the NAPC increases obviously with the damage developing. Because the damages are different for each element, there are differences among the NAPC results. For damage assessment, the relative change of NAPC is calculated as shown in Figure 25b. In the early stage of development of the concrete crack, the index change is up to 10%, sensitive enough to identify the damage happening, while it is over 50% for the larger damage. However, the detailed investigation of the damage sensitivity will be a future work, especially to detect initial damages that occur at the real structures through their service lives.

The resonant frequency is often taken as a useful index to identify structural damage. To explain the sensitivity of the proposed method, the resonant frequency of the first bending mode has also been extracted, namely, 33.25 Hz, 32.38 Hz, 30.42 Hz, 29.33 Hz and 28.87 Hz for the five cases. The relative change is 2.6%, 8.5%, 11.8% and 13.2% for Case D1, D2, D3 and D4. Considering the results in Figure 25b, the damage sensitivity of NAPC is much larger than the resonant frequency, which has also proved that the NAPC is a good local damage index.

#### 5.2.4. Neutral Axis Depth

**Figure 26:**
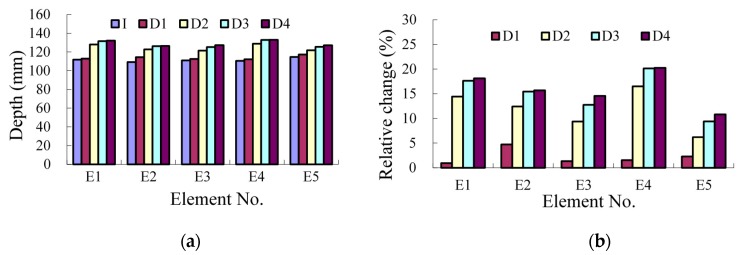
Results of neutral axis depth: (**a**) absolute value; and (**b**) relative change.

The neutral axis depth can be calculated by taking the NAPC results (Table 7) and the vertical distance between the two sensors (Table 8) into Equation (10), as shown in Figure 26a and Table 9. The results show the trend that the neutral axis moves towards the compressive zone with as the concrete cracks develop within the tensile zone. However, the relative change of the neutral axis is about 50% smaller than that of the NAPC (Figure 25b) as shown in Figure 26b.

The neutral axis depth has also been obtained from the static tests (Table 10) for further verification of accuracy of the proposed method. Table 11 shows results of the comparison between the neutral axis depth results obtained from the dynamic and the static tests. From the results, the smallest difference, not over 2.5%, is present at Case I, indicating the excellent accuracy of the proposed dynamic method. However, the difference increases greatly after the cracks happening. Another trend is that the neutral axis depth obtained with the proposed dynamic method is about 20% smaller than that from the static tests. The likely reason is that the crack closes after the static load is unloaded. Meanwhile the dynamic load is not large enough to make the crack open. Therefore, the actual neutral axis location is different at the dynamic and static loading cases. 

## 6. Conclusions and Remarks

In this paper, a new method is proposed to determine neutral axis position and identify local damage for flexural structures using modal macro-strain. Based on the research, the following conclusions can be drawn:

(1) The linear relationships also exist as the traditional plane section assumption when the modal macro-strain (MMS) of the first bending mode is applied for long-gauge monitored element.
(2) The neutral axis position can be determined with high accuracy by using the MMS based method. For example, the neutral depth assessment error is not larger than 2.5% for the Case I of RC beam experiment and all the cases of the steel beam experiment. Meanwhile, the proposed method offers excellent stability because all the correlation coefficients of the fitting lines are larger than 0.99 for all the tests reported in this paper.
(3) The neutral axis position coefficient (NAPC) is potentially a good indicator for local damage identification, such as concrete crack, as it presents an excellent damage sensitivity and assessing robustness even under 5% noise. The two sensors used to measure dynamic macro-strain need to be installed at each side of the neutral axis to obtain the best sensitivity.

However, there is still some work to be done in the future to make the proposed method more useful and applicable. Firstly, different types of beam sections and actual bridge models should be investigated to further verify the proposed method as the studied models in this paper are too simple. Secondly, field tests should be implemented to verify the proposed method under live traffic loads. Thirdly, except concrete crack some other damages, such as steel corrosion, should be considered to verify the effectiveness of the proposed method. In a word, there is a lot of research to be implemented to apply the proposed new method. Therefore, the work in this paper is just the beginning. Due to the excellent properties, a positive future can be expected, especially for the application of the proposed method in concrete infrastructures.

## Figures and Tables

**Table 1:** The damage case for FE models.

Damage Case	I	D1	D2	D3
Stiffness loss (%)	0	30	70	100

**Table 2:** The results of neutral axis position coefficient (NAPC).

Damage Case	I	D1	D2	D3
Modal analysis	1.007	1.047	1.144	1.334
Static analysis	1.000	1.042	1.141	1.330
Relative deviation (%)	0.7	0.5	0.3	0.3

**Table 3:** Comparison results of damage sensitivity (%).

Damage Case	Resonant Frequency (Bending Mode)					Neutral Axis Depth	NAPC	Strain Mode [16]	Curvature Mode [17]
	First	Second	Third	Fourth	Fifth				
D1	−0.1	0.0	−0.1	0.0	0.0	1.9	4.0	4.2	2.3
D2	−0.3	0.0	−0.3	0.0	−0.1	6.3	13.6	15.3	8.5
D3	−0.8	0.0	−0.8	0.0	−0.4	13.9	32.5	39.2	22.2

**Table 4:** Damage cases for the steel beam test.

Damage Case	I	D1	D2	D3	D4	D5	D6	D7	D8
Damage quantity of E_3_ (%)	0	3	5	15	18	18	18	18	18
Damage quantity of E_7_ (%)	0	0	0	0	0	3	5	15	18

**Table 5:** The results of neutral axis depth with modal analysis (mm).

Damage Case	I	D1	D2	D3	D4	D5	D6	D7	D8
E_1_	50.0	50.2	50.2	50.2	50.3	50.3	50.1	50.4	50.3
E_2_	49.5	49.7	50.0	49.8	49.8	49.7	49.6	49.9	49.8
E_3_	49.9	51.5	52.3	56.3	57.4	57.4	57.3	57.4	57.4
E_4_	50.0	50.2	50.3	50.3	50.7	50.8	50.5	50.8	50.7
E_5_	49.7	49.8	49.8	49.7	49.8	49.8	49.8	49.8	49.8
E_6_	50.1	50.3	50.5	50.3	50.4	50.2	50.2	50.5	50.3
E_7_	49.7	49.8	49.7	49.7	49.8	51.5	52.1	56.6	57.4
E_8_	50.6	50.8	50.7	50.8	50.9	50.8	50.9	51.5	51.5

**Table 6:** The relative difference compared with static tests (%).

Damage Case	I	D1	D2	D3	D4	D5	D6	D7	D8
E_1_	0.6	0.5	−0.2	0.8	0.5	1.0	−1.1	−0.6	−0.3
E_2_	−0.4	0.1	0.2	0.7	0.0	0.4	−1.2	−0.7	−0.2
E_3_	0.5	0.5	−0.1	1.8	1.2	1.7	−0.2	0.3	0.8
E_4_	−0.7	0.1	0.1	0.7	0.1	0.7	−1.4	−0.2	−0.1
E_5_	1.0	1.5	1.0	2.2	1.7	2.6	0.6	0.5	1.2
E_6_	0.3	1.0	1.1	1.7	1.4	1.5	−0.3	0.8	0.9
E_7_	0.3	1.0	0.0	1.0	0.5	1.7	−0.5	0.4	0.5
E_8_	1.0	1.8	1.2	2.2	1.8	2.4	0.8	1.8	1.7

**Table 7:** The results of NAPC.

Damage Case	I	D1	D2	D3	D4
E_1_	1.007	1.026	1.347	1.439	1.454
E_2_	1.023	1.126	1.318	1.403	1.410
E_3_	1.020	1.048	1.233	1.321	1.371
E_4_	1.068	1.103	1.511	1.635	1.640
E_5_	1.069	1.121	1.216	1.300	1.339

**Table 8:** The vertical distance between the two sensors (mm).

**Element Number**	E_1_	E_2_	E_3_	E_4_	E_5_
**Distance**	223	216	220	214	222

**Table 9:** The results of neutral axis depth with the proposed method (mm).

Damage Case	I	D1	D2	D3	D4
E_1_	112	113	128	132	132
E_2_	109	114	123	126	126
E_3_	111	113	121	125	127
E_4_	111	112	129	133	133
E_5_	115	117	122	125	127

**Table 10:** The results of neutral axis depth with the static method (mm).

Damage Case	I	D1	D2	D3	D4
E_1_	111	150	155	156	166
E_2_	110	145	149	149	161
E_3_	112	149	152	153	166
E_4_	112	149	153	153	161
E_5_	115	149	152	153	160

**Table 11:** The comparison results with the static method (%).

Damage Case	I	D1	D2	D3	D4
E_1_	1.2	−24.8	−17.6	−15.6	−20.5
E_2_	−1.1	−21.0	−17.7	−15.6	−21.3
E_3_	−0.7	−24.5	−20.3	−18.3	−23.2
E_4_	−2.2	−24.5	−16.0	−13.3	−17.4
E_5_	1.0	−21.1	−20.1	−18.2	−20.8
